# Estimating the impact of changes in HbA_1c_, body weight and insulin injection regimen on health related quality-of-life: a time trade off study

**DOI:** 10.1186/s12955-016-0411-0

**Published:** 2016-01-22

**Authors:** Martin Ridderstråle, Lyndon Marc Evans, Henrik Holm Jensen, Mette Bøgelund, Marie Markert Jensen, Åsa Ericsson, Johan Jendle

**Affiliations:** Steno Diabetes Center, Gentofte, Denmark; University Hospital Llandough, Cardiff, UK; Incentive, Holte, Denmark; Novo Nordisk, Søborg, Denmark; Novo Nordisk, Malmö, Sweden; Faculty of Health Sciences and Medicine, Örebro University, Örebro, Sweden; Endocrine and Diabetes Center, Karlstad Hospital, Karlstad, Sweden

**Keywords:** Time trade off study, Health related quality of life, HbA1c level, Body weight, Insulin injection regimen, Type 2 diabetes, Utility values

## Abstract

**Background:**

There are limited data on the potential short-term benefits associated with reductions in HbA_1c_ levels, and understanding any immediate improvements in health related quality-of-life (HRQoL) through better glycaemic control may help inform diabetes management decisions. This time-trade-off (TTO) study investigated the short-term impact on HRQoL associated with three different aspects of diabetes management; HbA_1c_ change, body weight change, and the complexity of treatment regimen.

**Methods:**

The study was designed in three stages: Stage 1) Qualitative telephone interviews with people with type 2 diabetes (T2D) in Denmark who had experienced a decrease in their HbA_1c_ level. Stage 2) A validation survey with people with T2D in Denmark to obtain quantifiable knowledge on the short-term effects of a change in HbA_1c_ levels. Stage 3) TTO survey using health states based on results from stage 2. Respondents were either adults with T2D (Sweden) or from the general public (UK and Denmark) and were separately asked to evaluate seven health states through an internet-based survey.

**Results:**

Results from 4060 respondents were available for the TTO analysis (UK *n* = 1777; Denmark *n* = 1799, Sweden *n* = 484). ‘Well-controlled diabetes’ was associated with utilities of 0.85–0.91 and ‘not well-controlled diabetes’ with utilities of 0.71–0.80 in all countries. Difference in utilities per HbA_1c_ percentage point was smallest in Sweden and largest in Denmark (between 0.025–0.034 per HbA_1c_ percentage point respectively). The treatment management health state associated with the lowest disutility was the once-daily insulin regimen. The disutility associated with per kg of weight change ranged from 0.0041–0.0073.

**Conclusions:**

Changes in HbA_1c_ levels, insulin regimen and body weight are all likely to affect HRQoL for patients with T2D. A change in HbA_1c_ is likely to have a short-term impact in addition to the effect on the development of long term diabetes complications. A treatment which has a simple regimen with fewer injections, and/or the need for less planning, and that causes weight loss or less weight gain, compared with other treatments, will have a positive impact on HRQoL.

**Electronic supplementary material:**

The online version of this article (doi:10.1186/s12955-016-0411-0) contains supplementary material, which is available to authorized users.

## Background

The long-term effects of changes in 3-month average plasma glucose, reflected in the HbA_1c_ level, are well documented for patients with diabetes [1–5]. Since therapeutic interventions reduce HbA_1c_ levels, there may also be some immediate improvements in health related quality-of-life (HRQoL) related to the improved glycaemic control e.g. reduction in symptoms such as feeling tired or thirsty. Understanding these possible relationships could help inform management decisions.

The majority of health economic evaluations for diabetes are based on the association between glycaemic control and the risk of developing long-term complications, especially the risk for cardiovascular complications. There are very few data on health utility values for short-term improvements in symptoms associated with improved glycaemic control independent of long-term complications [6].

Time trade off (TTO) is an accepted method to elicit health related utility values for different health states. The preference for one health state compared with another can be quantified as the difference in elicited utility values between the two health states, for example, different weight effects or different treatment regimens. While some attributes may be relatively simple to convey in a health state description, such as weight change or the number of injections per day, the short-term effects of different levels of glycaemic control in health state descriptions may be harder to describe in a concise and accurate manner for the general non-diabetic population. Conveying the short-term effects of different levels of glycaemic control in health state descriptions requires knowledge of the symptoms and changes in symptoms that might be associated with changes in HbA_1c_ levels.

The aim of this TTO study was to investigate the short-term impact on HRQoL associated with three different aspects of diabetes management; HbA_1c_ change, body weight change, and the complexity of treatment regimen, using a methodology that allows comparison between their associated health states.

## Methods

The study was designed in three stages with the overall objective to understand how changes in body weight, multiple daily insulin injection treatment regimens and HbA_1c_ affect HRQoL on a daily basis. The first two stages aimed to describe appropriate health states for further investigation via the TTO methodology used in stage 3.

Prior to commencing the study, diabetologists with experience with the TTO methodology were identified and invited to form an expert steering group (ESG) to advise at all stages of the study.

The first stage involved qualitative telephone interviews with people with type 2 diabetes (T2D) who had experienced a decrease in their HbA_1c_ level. This was conducted to gain a preliminary understanding of the short-term effects and experiences associated with a drop in HbA_1c_, as well as confirmation of results from previous studies about weight and regimen preferences [7]. The outcome of this was guidance for developing the subsequent validation survey in stage 2. The validation survey aimed to provide clear and quantifiable knowledge, from a larger number of patients with diabetes, on the short-term effects of a change in HbA_1c_ levels. The output from this stage was description of two health states; one associated with a high HbA_1c_ level and one associated with a low HbA_1c_ level. Following review by the ESG, the resulting data were used to obtain a description of symptoms to be included in the TTO survey (stage 3).

### Stage 1 – telephone interviews

Qualitative telephone interviews were undertaken in Denmark in May 2014 to explore the experience of individuals with T2D experiencing a decrease in HbA_1c_. Respondents were recruited for telephone interviews in collaboration with Steno Diabetes Center (Gentofte, Denmark). In addition to identifying individuals who had achieved a decrease in HbA_1c_, simple questions about body weight change and treatment regimens were included e.g. “Have you lost weight?” and “What is your current treatment regimen?” An interview guide ensured consistency in approach. All respondents gave informed consent to participate.

### Stage 2 – validation survey

The validation survey was conducted in Denmark in June 2014 using an online panel of individuals with T2D who had previously agreed to participate in internet-based surveys. In an attempt to gain a representative sample of the general T2D population various channels, including telephone interviews, face-to-face interviews and web banners, were used for recruitment. For inclusion in the validation survey respondents were required to be adults diagnosed with T2D, receiving medication, and knowing their current HbA_1c_ level. Respondents were offered a nominal remuneration of less than 1€ to compensate for their time.

Questions focused on the respondent’s last measured HbA_1c_ (current level) and how often they experienced specific symptoms using ranking on a 5-point scale (always, often, sometimes, rarely, never). The selected symptoms were based on the results of the stage 1 telephone interviews and included; tiredness/lack of energy; fatigue/extreme tiredness; headache; excessive thirst; dry mouth; frequent urination during the day or night; blurred vision; slow healing of wounds; poor memory; mood swings; difficulty concentrating. Psychological impact of poor glycaemic control was also measured using the validated Problem Areas in Diabetes 5-item scale (PAID-5) [8]. This overall methodology has been used to define health states in a previous diabetes study [9].

In order to identify any changes in symptoms associated with a change in HbA_1c_, respondents were also asked to recall prior HbA_1c_ levels (previous level) either at the time of diagnosis (treatment initiation with oral antidiabetic agent (OAD)); at the time when they started on insulin (basal-only insulin) or, at the time when they progressed to bolus insulin (basal–bolus regimen) and to recall how often they experienced the various symptoms at this time. HbA_1c_ levels were recorded in intervals of 0.5 % from <6 % and up to ≥12 %. Respondents reporting an HbA_1c_ level above 12 % were assigned the value of 13 % for analytical purposes. The difference between the highest and lowest reported HbA_1c_ levels for those respondents reporting the greatest change were compared, as well as the frequencies of symptoms associated with the highest and lowest HbA_1c_ levels. Physical and psychological symptoms were further investigated by dividing respondents into three groups according to HbA_1c_ change: >2 %-points, 1-2 %-points, <1 %-point. The difference in symptom frequency scores from the time with a high self-reported HbA_1c_ to the time of a low self-reported HbA_1c_ was calculated to give an impression of whether the symptom was experienced more often with the high HbA_1c_ level than with the low HbA_1c_ level. These comparisons were undertaken using descriptive statistics and regression analysis.

### Stage 3 – TTO survey

The health states used in the TTO survey were developed based on the results of the telephone interviews and validation survey, and following review and consultation with the ESG. Each symptom was evaluated for relevance for inclusion in health state descriptions on the basis of a significant association between the HbA_1c_ level and the symptom frequency. A test health state was used to familiarise the respondents with the TTO exercise, as outlined in Additional file 1: Table S1. These results were not used in the final analysis.

For change in HbA_1c_, two health states were developed; ‘*not well-controlled diabetes’* ‘and ‘*well-controlled diabetes*’. The ‘*not well-controlled diabetes*’ state reflected the symptoms of the group having experienced the highest HbA_1c_ levels (average 11.6 %). The ‘*well-controlled diabetes’* state reflected the symptoms of all the groups having experienced any change in HbA_1c_ with a lowest average HbA_1c_ level of 7.4 %. Both health states are outlined in Table 1.

**Table 1:** HbA_1c_ health states derived from the validation survey

Well-controlled diabetes	Not well-controlled diabetes
Imagine that you have diabetes as described.	Imagine that you have diabetes as described.
Imagine that you succeed in keeping your treatment plan. Your doctor has told you that your average blood sugar is on target. Therefore:	However, for some time you have had trouble following your treatment plan. Your doctor has told you that your average blood sugar is higher than it should be. As a consequence:
• You rarely feel excessive thirst and rarely have to urinate more often, neither during the day nor during the night. • You rarely drink a lot. • A minor problem for you is feeling scared and depressed thinking about living with diabetes. • Keeping your blood sugar at a good level requires constant focus on your lifestyle.	• You sometimes feel excessive thirst and have to urinate more often, during both the day and the night. • You sometimes also drink a lot. • Sometimes you feel tired and without energy. • A problem for you is feeling scared and depressed thinking about living with diabetes. • You wish your average blood sugar were lower, but it would require several lifestyle changes and you do not want diabetes to control your life.
Imagine what it would be like for you to live with diabetes every day for the rest of your life.	Imagine what it would be like for you to live with diabetes every day for the rest of your life.

For change in insulin regimen, health states were developed for ‘*well-controlled diabetes’* with differences in respect of regimen and the need for daily planning of injection times, dose adjustment or monitoring of calorie intake. These covered injection once-daily, injection twice-daily, injection twice-daily including daily planning, and injection four-times-daily including daily planning (Table 2). For change in body weight, health states were developed for ‘*well-controlled diabetes’* with different weight gains or losses; 5 kg gain, 2 kg gain, 5 kg loss and 2 kg loss (Table 3).

**Table 2:** Insulin regimen health states

Health state	Description
Injection twice daily including planning	• Imagine that you have well-controlled diabetes as described. • In order to control your blood sugar level, you must give yourself two insulin injections every day using two different pens. One injection should be taken in the morning. The other should be taken in connection with a large meal. • The second insulin dose should fit the type and size of your meals as well as your activity level. Therefore you have to plan either to eat roughly the same type and size of meals or ad- just your insulin doses every day. • Imagine what it would be like for you to live like this every day for the rest of your life.
Injection four times daily including planning	• Imagine that you have well-controlled diabetes as described. • In order to control your blood sugar level, you must give yourself four insulin injections every day using two different pens. • One injection should be taken independent of meals at the same time every day. Using the other pen you give yourself insulin injections with each of your 3 largest meals during the day (breakfast, lunch and dinner). • These three insulin doses should fit the type and size of your meals as well as your activity level. Therefore you have to plan either to eat roughly the same type and size of meals or ad- just your insulin doses accordingly. • Imagine what it would be like for you to live like this every day for the rest of your life.
Injection once daily	• Imagine that you have well-controlled diabetes as described. • In order to control your blood sugar level, you must give yourself one insulin injection every day. • The injection must be taken at approximately the same time every day. • Imagine what it would be like for you to live like this every day for the rest of your life
Injection twice daily	• Imagine that you have well-controlled diabetes as described. • In order to control your blood sugar level, you must give yourself two injections every day using two different pens (one of which is insulin). • Both injections must be taken at approximately the same time every day. • Imagine what it would be like for you to live like this every day for the rest of your life.

**Table 3:** Change in body weight health states

Health state	Description
Gain 5 kg	• Imagine that you have well-controlled diabetes as described. • The doctor changes your medication and you gain 5 kg without changing your lifestyle. • Imagine what it would be like for you to live like this every day for the rest of your life.
Gain 2 kg	• Imagine that you have well-controlled diabetes as described. • The doctor changes your medication and you gain 2 kg without changing your lifestyle. • Imagine what it would be like for you to live like this every day for the rest of your life.
Lose 2 kg	• Imagine that you have well-controlled diabetes as described. • The doctor changes your medication and you lose 2 kg without changing your lifestyle. • Imagine what it would be like for you to live like this every day for the rest of your life.
Lose 5 kg	• Imagine that you have well-controlled diabetes as described. • The doctor changes your medication and you lose 5 kg without changing your lifestyle. • Imagine what it would be like for you to live like this every day for the rest of your life.

Respondents were asked to evaluate seven health states (as shown in Table 4), using TTO methodology as previously described [10–12]. To make the trade-offs as realistic as possible, time horizons were based on each respondent’s projected life expectancy obtained using the country, age and gender of the respondent and the most recent World Health Organization data [13]. A starting utility value of 0.6 was used; utility values for each respondent were determined to a precision of 0.05. To identify the point of indifference (i.e. when both health state options were equally acceptable), the respondents were asked the TTO question repeatedly (up to 6 times), whilst varying the number of years living in full health (as illustrated in Additional file 1: Figure S1). An example TTO question, as it appeared on the screen, is shown in Additional file 1: Figure S2.

**Table 4:** Health states for each respondent

Health state	Each respondent evaluated
Warm-up health state	Diabetes derived from previous work. Not used.
HbA1c	Well-controlled diabetes
	Not well-controlled diabetes
	(The order of the two health states were randomised)
Weight	1 health state associated with weight gain (2 kg or 5 kg randomised)
	1 health state associated with weight loss (2 kg or 5 kg randomised)
	(The order of the two health states were randomised)
Regimen	Two randomly selected out of the four health states (i.e. injection twice daily including planning, injection four times daily including planning, injection once daily, injection twice daily

Data were collected in Sweden, Denmark and the United Kingdom (UK) through an internet-based survey. In Sweden, respondents were adults diagnosed with, and receiving medication for, T2D. In the UK and Denmark, respondents were members of the general public. These differences in sample collection are due to specific requirements from the individual countries’ health technology assessment authorities. All respondents had previously agreed to participate in internet-based surveys and various channels were used for recruitment to attempt to gain a representative internet panel. Only individuals aged ≥18 years were approached and took part at their discretion; anonymity was preserved throughout. As with the validation survey, respondents were offered a nominal remuneration of less than 1€ to compensate for their time. The functionality of the questionnaire was validated in a pilot study. In addition to questions related to the TTO exercise, respondents provided data on demographics and employment status. Respondents with diagnosed diabetes were also asked to provide further information regarding the duration of their diabetes and their current medication regimen.

Respondents who chose not to trade any lifetime (i.e. preferred to live in the health state described rather than in full health in all TTO questions, illustrated in Additional file 1: Figure S1), or were willing to trade a very high proportion of their remaining lifetime to be restored to full health (i.e. preferred to live in full health in all TTO questions, illustrated in Additional file 1: Figure S1, were carefully investigated. Such respondents were excluded if they refused to trade time on ethical or religious grounds, or if they did not understand the questions. However, those who believed the health state manageable or who stated desire to live as long as possible due to obligations (e.g. caregivers) were retained.

#### Statistical analysis

All statistical analyses were performed using SAS® (version 9.4) statistical software. A utility value was assigned to each health state based on each individual response, derived from the midpoint of the indifference interval. The average utility value was calculated for each health state, and utility differences for the relevant health states were calculated.

As the response distribution was unknown but suspected to be non-normal, non-parametric bootstrapping was used to simulate standard errors and confidence intervals (CIs) for the mean TTO values. This method estimates the parameter’s distribution by repeatedly resampling the original data set with replacement [14–16]. A total of 10,000 iterations were performed. The difference between utility values for health states was deemed to be significantly different from zero if the confidence intervals obtained by bootstrapping around this difference remained above zero.

In the main analysis those with the 2.5 % lowest and the 2.5 % highest utility differences were excluded. This objective cut-off was chosen as a way to increase stability of the results without having to apply different subjective cut-off points for each of the three health states. Sensitivity analyses were also undertaken applying different cut-off criteria.

#### Estimation of HRQoL impact per HbA_1c_ point

The results of the validation study suggested that the greater the reduction in HbA_1c_, the greater the effect on symptom frequency in the health states. A significant association was seen between changes in HbA_1c_ levels and changes in all physical and the majority of the psychological short-term symptoms included in the TTO health states.

To allow interim utility values to be derived, an assumption was made that utility changes were linear between the ‘not-well controlled’ and ‘well controlled’ health states, defined by a 4.2 % difference in HbA_1c_ (11.6 %–7.4 %). Therefore the utility difference between health states for each individual was divided by 4.2 to calculate the average increase in utility per HbA_1c_ percentage-point change.

As the 4.2 % difference was derived from self-reported HbA_1c_ levels stated in interval categories (with censoring at each end), sensitivity analyses were also performed using various different cut-off criteria and applying uncertainty in the HbA_1c_ difference between the two health states.

#### Estimation of HRQoL impact from insulin regimen features

The impact on HRQoL of different insulin regimens was derived by calculating the difference in utility between the insulin regimen health states and well controlled diabetes. The resulting difference in values thus reflects the effect of the changed feature in treatment regimen description alone, with no confounding influence from the estimated disutility associated with having diabetes.

#### Estimation of HRQoL impact of body weight changes

The impact on HRQoL due to body weight changes was derived by calculating the difference in utility between each of the two weight-related health states presented to each respondent. Each respondent evaluated two of four different health states for body weight; one implying weight loss of either 2 kg or 5 kg and another implying weight gain of either 2 kg or 5 kg.

Subsequently, an average utility per kilogram weight change was calculated and in the same way, the utility per change in BMI units was also derived.

## Results

### Stage 1 – telephone interviews

In total, eight individuals with T2D who had experienced a drop in HbA_1c_ completed telephone interviews (88 % male and average age of 72 years). Results of the telephone interviews confirmed that even though patients aspire to lose weight, most were not willing to change their lifestyle to obtain weight loss. Examples of short-term symptoms that might be associated with high HbA_1c_ levels such as thirst, dryness of the mouth, increased urination (both volume and frequency), tiredness (ranging from little to extreme) and psychological issues were identified for inclusion in the validation survey.

### Stage 2 – validation survey

A total 268 out of 298 respondents could recall both their current and previous HbA_1c_ levels. The majority of respondents were male (72 %), with average age of 66.5 (7.7) years and average 11 years (6.9) duration of diabetes. The average current HbA_1c_ level was 7.5 %. The average change in HbA_1c_ was 2.0 %-points. Respondents who had experienced a change in HbA_1c_ of >2 %-points had an average high HbA_1c_ of 11.6 %. The average lowest HbA_1c_ for all respondents having experienced a change was 7.4 %; thus giving a difference of 4.2 %-point (11.6 %–7.4 %) between these two groups.

The association between changes in the frequency of experiencing symptoms and change in HbA_1c_ levels was significant for the majority of symptoms, as outlined in Table 5 and detailed in Additional file 1: Figure S3–S8.

**Table 5:** Results from the validation survey for the association between change in HbA_1c_ levels and change in frequency of short-terms symptoms

Symptom	Results
Excessive thirst	Significant association between change in the frequency of experiencing excessive thirst and change in HbA_1c_ (*p* < 0.0001).
Frequent urination (day and night)	Significant association between change in the frequency of experiencing frequent urination and change in HbA_1c_ (*p* < 0.0001, *p* = 0.0005 for day and night respectively)
Fatigue/extreme tiredness and tiredness and lack of energy	Significant association between change in the frequency of experiencing fatigue symptoms and change in HbA_1c_ (*p* < 0.0001 and *p* = 0.0001 for tiredness/lack of energy and fatigue/extreme tiredness respectively).
Psychological	
Feeling scared when thinking about living with diabetes	Significant association between feeling scared…” and change in HbA_1c_ (*p* = 0.0328). The p value for the association between “feeling depressed…” and change in HbA_1c_ was *p* = 0.0557.
Feeling depressed when thinking about living with diabetes	
Wishing to reduce HbA_1c_	The higher the current HbA_1c_ level, the more the respondents wanted to reduce their HbA_1c_ level (*p* < 0.0001).

### Stage 3 – TTO survey

Total number of respondents was 2159 in the UK, 2420 in Denmark and 723 in Sweden. Of these, valid responses were available from 1777 respondents in the UK, 1799 respondents in Denmark and 484 respondents in Sweden. Responses were excluded from 1242 respondents, either because they were only partly complete (*n* = 702), respondents refused to trade on ethical or religious grounds (*n* = 316), or if respondents failed a test question (i.e. those who preferred living a shorter time with diabetes than living for longer in full health) (*n* = 224). The profile of respondents included in the final sample is given in Table 6.

**Table 6:** Final sample respondents’ profile

	UK	Denmark	Sweden
	*N* = 1777^a^	*N* = 1799^a^	*N* = 484^b^
Male (%)	50	50	63
Age, years (mean)	43.5 (14.5)	45.5 (15.8)	63.8 (9.2)
BMI kg/m^2^ (mean)	26.1 (5.7)	26.2 (5.4)	30.1 (5.3)
Treatment with oral anti-diabetic drugs (%)	-	-	87 %
Insulin treatment (%)	-	-	34 %
Diabetes duration, years (mean)	-	-	9.5 (7.0)
Duration of insulin treatment (mean years)	-	-	7.5 (6.6)
Currently employed (%)	59	52	31

^a^Sample of the general population ^b^Adults with Type 2 diabetes

#### HbA_1c_ health states

**Fig. 1:**
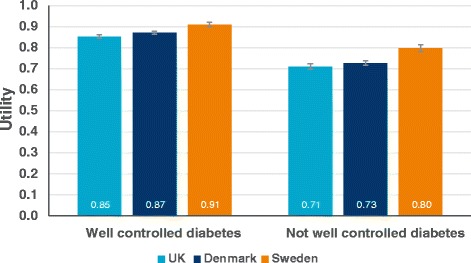
Utilities for well-controlled and not well-controlled diabetes health states. The error bar denotes the 95 % confidence interval derived from bootstrapping

The results for the utility values of the two HbA_1c_ health states are presented in Fig. 1. As expected, ‘well-controlled diabetes’ was associated with higher utility than ‘not well-controlled diabetes’ in all countries with the difference being significantly different from zero (UK 0.139, 95 % CI 0.132, 0.147; Denmark 0.141, 95 % CI 0.134, 0.149; Sweden 0.106, 95 % CI 0.094, 0.119). In Sweden, however, respondents evaluated both health states with higher utility compared to the respondents in the UK and Denmark. The difference between the values for both health states was also smaller in Sweden. The difference in utilities per HbA_1c_ percentage point (Table 7) was smallest in Sweden and largest in Denmark (utility difference between 0.025–0.034 per HbA1c percentage point respectively).

**Table 7:** Utility results for the UK, Denmark and Sweden

	UK^a^			Denmark^a^			Sweden^b^		
	Utility value	Low (2.5 %)	High (97.5 %)	Utility value	Low (2.5 %)	High (97.5 %)	Utility value	Low (2.5 %)	High (97.5 %)
HbA_1c_ change (per percentage point)	0.033	0.031	0.035	0.034	0.032	0.035	0.025	0.022	0.028
Weight change									
Per kg	0.0073	0.0065	0.0081	0.0041	0.0036	0.0046	0.0068	0.0057	0.0081
Per BMI-unit	0.021	0.018	0.023	0.012	0.011	0.014	0.021	0.017	0.024
Insulin regimen									
Once daily vs twice daily	0.020	0.010	0.031	0.021	0.014	0.027	0.015	0.0001	0.029
Once daily vs twice daily planning	0.046	0.035	0.058	0.043	0.036	0.051	0.038	0.021	0.054
Once daily vs four times daily planning	0.070	0.057	0.082	0.084	0.075	0.093	0.109	0.086	0.133
Twice daily vs twice daily planning	0.026	0.014	0.037	0.022	0.015	0.030	0.023	0.006	0.040
Twice daily vs four times daily planning	0.049	0.037	0.062	0.064	0.055	0.072	0.095	0.071	0.118
Twice daily planning vs four times daily planning	0.023	0.010	0.037	0.041	0.031	0.051	0.071	0.047	0.096

^a^Sample of the general population ^b^Adults with Type 2 diabetes

Sensitivity analysis showed that the results were not overly sensitive and that the cut-off values used were appropriate (Table 8).

**Table 8:** Sensitivity analyses of HbA_1c_ difference between health states “well-controlled” and “not well-controlled” diabetes using different cut-off criteria

	2 %, 5 % and 10 % cut off criteria			Different cut-off criteria (2.5 % cut off)		
	2 % cut off	5 % cut off	10 % cut off	Diff. 3.5 %	Diff. 4.2 %	Diff. 4.7 %
UK	0.144	0.139	0.130	0.038	0.033	0.029
Denmark	0.147	0.141	0.131	0.038	0.034	0.025
Sweden	0.110	0.106	0.098	0.029	0.030	0.023

#### Insulin regimen health states

**Fig. 2:**
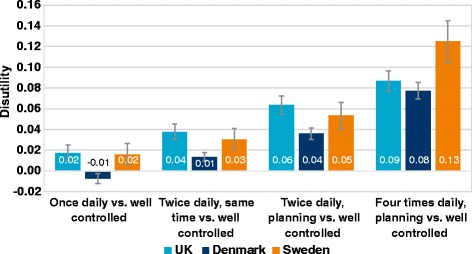
Disutility associated with insulin regimen compared to well-controlled diabetes. Error bars denote 95 % confidence intervals

Results for the different insulin regimens compared to the health state ‘well controlled diabetes’ are shown in Fig. 2. In all three countries, the health state associated with the lowest disutility (the best health state) was the once-daily insulin regimen. Regimens with additional injections and/or patients having to plan around injections and meal-times increased the disutility. The health state with the highest disutility (the worst health state) was the basal–bolus health state with four daily injections and the associated planning. In Denmark, the difference between the once-daily insulin regimen health state and ‘well controlled diabetes’ was not significantly different from zero (−0.01, 95 % CI −0.012, −0.003).

Selected differences between the four different regimen health states (Table 7) suggested that one additional injection (without planning) was valued in the range of 0.015–0.021. Additional planning with a twice daily injection regimen reduced the utility value by between 0.022–0.026. Furthermore, the difference between a basal–bolus regimen of four injections and once-daily planned regimen was valued between 0.070–0.109. The basal–bolus regimen had the greatest negative impact on HRQoL compared with the other regimens.

##### Body weight health states

**Fig. 3:**
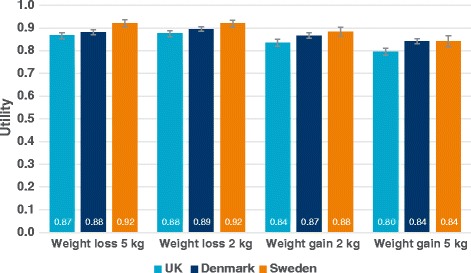
Utilities for weight gain or loss health states. Error bars denote 95 % confidence intervals

Figure 3 illustrates the utilities assigned to the four body weight health states. As expected, results in all three countries suggested that respondents would prefer to lose rather than gain weight. Gaining 5 kg was valued in the range of 0.80–0.84 whereas gaining 2 kg was valued in the range of 0.84–0.88. The ranges for losing 5 kg and losing 2 kg were similar (0.87–0.92 and 0.88–0.92 respectively).

The consolidated results by country are detailed in Table 7.

## Discussion

This study aimed to quantify the short-term HRQoL impact associated with change in three different aspects of diabetes management; HbA_1c_ level, weight change, and the complexity of treatment regimen using TTO methodology. TTO produces valuations based on decisions made under conditions of certainty and is an accepted method for preference valuations of health states by various health-technology assessment bodies (e.g. National Institute for Health and Care Excellence in the UK). In this TTO study, the trade-offs were individually based on the respondents’ age and life-expectancy, which may increase the relevance of the methodology and avoid some of the disadvantages of the fixed 10- or 30-year horizon used in other studies [11, 17].

Our results for HbA_1c_ showed a significant association between change in HbA_1c_ and frequency of experiencing hyperglycaemia related symptoms (frequent urination and excessive thirst). As expected, well-controlled diabetes was associated with higher utility than not well-controlled diabetes.

The difference between the two health states was significantly different from zero for each of the three countries; the population with diabetes from Sweden valued both health states higher (better) than the general populations from the UK and Denmark. Similar results have been observed in other studies [6] and suggest that people with experience of a disease give less value to its associated impacts on HRQoL than those who do not have the disease. The population with diabetes were also older on average (63.8) than the general populations from UK (43.5) and Denmark (45.5) which may have also influenced perceptions of HRQoL. There were also small differences between the values for the UK and Denmark which may have many causes, for example differences in each health care system and the way diabetes is viewed by society. Only two previous studies have analysed the relationship between HbA_1c_ levels and short term effects on HRQoL in T2D [18, 19]. Peyrot et al. [18] investigated the relationship between HbA_1c_ levels and HRQoL measured by the EQ-5D in adults with T2D and showed that a change in HbA_1c_ of −1.20 % was associated with an improvement in health utility of 0.03 (95 % CI 0.00, 0.07); our results are very similar with a 1 % change in HbA_1c_ being associated with an improvement in health utility of 0.025 in those respondents with T2D. The study by Koopmanschap [19] also found an association between HbA_1c_ and health utility in T2D although no direct estimate was reported in this study. A recent study by McQueen et al. [6] also found similar utility values (1 % increase in HbA1c was associated with a disutility of −0.03 [95 % confidence interval [CI]-0.049, −0.006]) for association between HbA_1c_ and health utility in T1D.

Several studies have shown that a complex treatment regimen can negatively impact adherence [20–22]. We aimed to substantiate data on the potential effects of complex insulin injection regimens in this study by looking at how these can affect HRQoL. Our results confirm that regimen has an impact on HRQoL and again, the respondents with diabetes in Sweden gave a higher value to each health state than the respondents from the general population in the UK and Denmark. The basal–bolus regimen had the greatest impact on HRQoL; the difference between a basal–bolus regimen of four injections and a once-daily planned regimen was 0.102 (0.007–0.109). Our results are similar to those reported recently in a TTO study designed to assess the HRQoL impact of flexible basal insulin doses [23]. The result showed that in the general population time-flexible basal insulin injections were associated with 0.016 and 0.013 higher utility compared with a fixed time of injection for basal-only and basal–bolus regimens, respectively. Once-daily injections also had higher utility compared with twice-daily injections for basal (0.039 and 0.042) and basal–bolus (0.022 and 0.021) regimens, for the general population and for those with diabetes, respectively.

Diabetes medications differ with respect to anticipated changes in body weight and we were interested to see how weight gain and weight loss may affect HRQoL. Our results suggest that the utility value per kg of body weight is largest when comparing weight gain of 5 kg with weight loss of 2 kg. The impact is higher than the combination of weight loss of 5 kg compared to weight gain of 2 kg implying that avoiding weight gain is more important than weight loss. These results are in alignment with other similar studies [24–26]. A recent study that aimed to estimate utility values for hypothetical health states associated with differences in weight and quality of life in Canadians with T2D showed for every decrease of 1 kg/m [2] BMI there was an associated increase in utility of 0.0171 (95 % CI: 0.0103, 0.0238) [26]. Our study showed a utility of 0.021 for each BMI unit lost in those respondents with T2D.

The key limitation of the methodology is the assumption that utility values change linearly as HbA1c and weight changes. This assumption allows the calculation of interim utility values and was made based on consultation with the clinical experts. Further limitations include those associated with internet-based surveys. Web-based methodology means that help cannot be offered if respondents have queries and with a lack of supervision, there is also a risk that some respondents may not spend adequate time considering their answers. We sought to minimise this through the survey design, ensuring a 10-s delay before proceeding to the next question. Additionally, the use of an internet-based survey may pose a selection bias, since only literate respondents with internet access can participate. However, the literacy rates and proportion of internet users in the three countries is high [27]. The potential for a long recall period in the study could also affect the accuracy of respondent reporting of symptoms. Lastly, by offering incentives to participate there is a risk of bias, however, the remuneration offered was small (<€1).

## Conclusion

Based on reported preference for different health states, these results suggest that changes in HbA_1c_ levels, insulin regimen and body weight are all likely to affect HRQoL for patients with diabetes. Furthermore, a change in HbA_1c_ is likely to affect some aspects of daily living in addition to the effect on the development of long term diabetes complications. These results suggest that a treatment which has a simple regimen with fewer injections, and/or the need for less planning, and that causes weight loss or less weight gain, compared with other treatments, will have a positive impact on HRQoL.

## Additional file

Additional file 1: Table S1 and Figures S1-S8. (DOCX 622 kb)
