# Supplementary material for: Estimating the impact of changes in HbA1c, body weight and insulin injection regimen on health related quality-of-life: a time trade off study
Source: Health Qual Life Outcomes. 2016 Jan 22;14:13. doi: 10.1186/s12955-016-0411-0 (PMC4722746; doi:10.1186/s12955-016-0411-0)
Supplement: Additional file 1: Table S1 and Figures S1-S8. — (DOCX 622 kb) [file 12955_2016_411_MOESM1_ESM.docx]

# Additional file 1

Table S1. Warm-up health state

| Diabetes |
| --- |
| Imagine that you have a health condition called diabetes:   - This means that your body cannot keep your blood sugar at a constant level. - To control this, you often follow a special diet, are careful about eating regularly and take steps to have something sweet nearby. - You take medication on a daily basis and sometimes need to plan your life around food and medicine. - You need to check your blood sugar from time to time. - You need to consider your diabetes when you are planning to exercise, travel, go out with friends and drive. - Your diabetes does not affect your work/study, and you don't have any problems looking after yourself. - You occasionally worry about the effects of your diabetes on your day-to-day life. - Your diabetes is well-controlled and you do not experience hypoglycemic episodes (hypos; too low blood sugar levels).   Imagine what it would be like for you to live with diabetes every day for the rest of your life. |

Figure S1. Deriving utility values using the TTO method


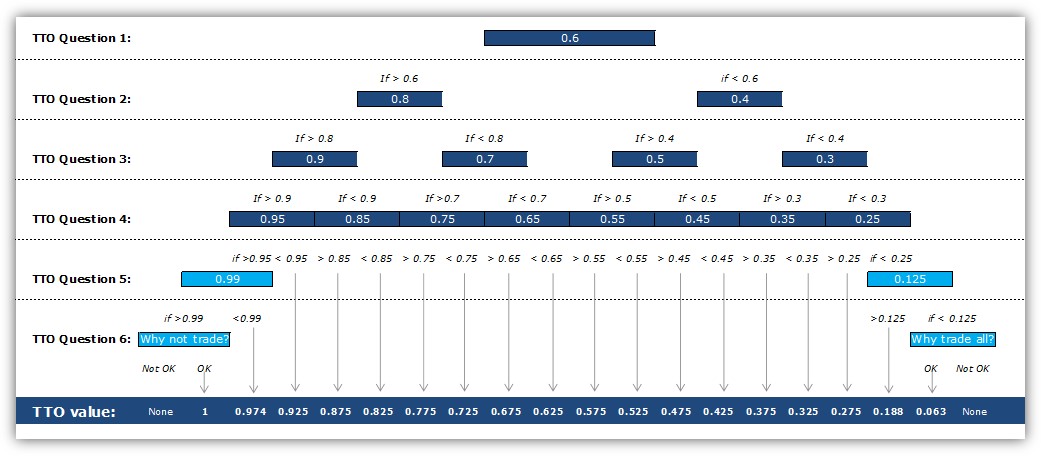


A starting utility of 0.6 was used in the TTO process and provided an indication of whether the respondent preferred to live in the health state in question (i.e. utility in this health state was >0.6) or in full health (i.e. utility in this health state was <0.6). Subsequent TTO questions were varied in accordance with this response. For example, if the value was >0.6, then the respondent was given the same trade off, but with a value of 0.8. The question was repeated up to six times. If a respondent preferred to live in full health in all TTO questions (i.e. questions 1–5), they were identified as ones who were willing to trade a very high proportion of their life to live in full health. Such respondents were then asked an additional question, namely, “Why trade all”. Conversely, if a respondent preferred to live in the described health state in TTO questions 1–5, they were identified as ones who were not willing to trade and asked an additional question (namely, “Why not trade”. All values were translated into years and months (based on the expected lifetime remaining), when posing the TTO questions to respondents.

Figure S2. Example of a TTO question (for 25 year old, female respondent)

a. The question is preceded by an introduction of the health state (in this case, “not well-controlled diabetes).


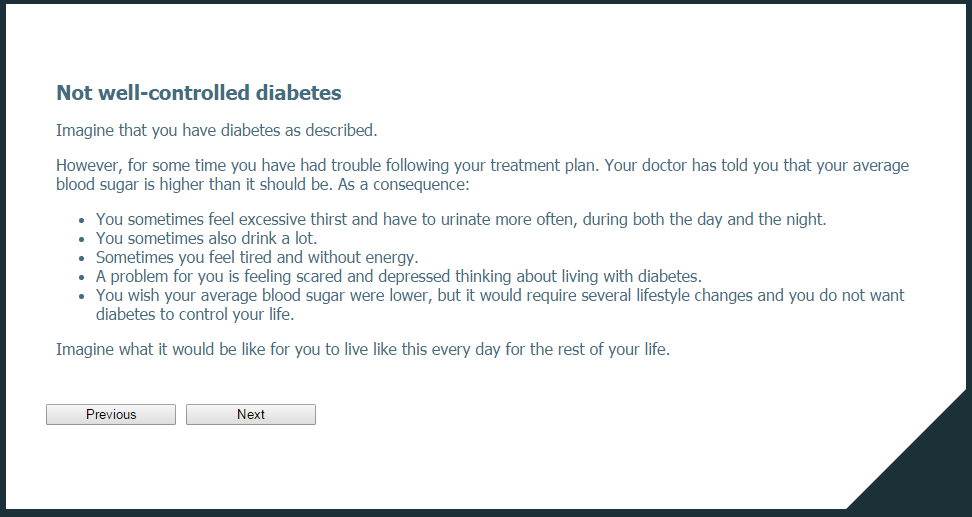


b. The introduction is followed by the first TTO question, where the time in full health is 0.6 times that in the health state in question.


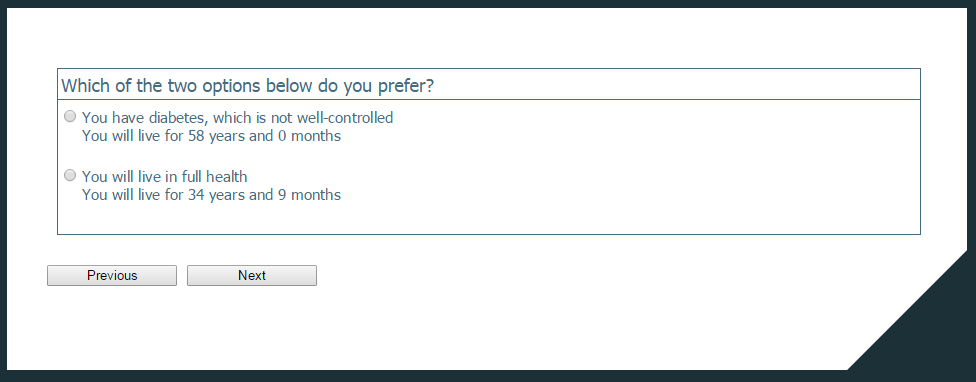


The years and months were derived, based on the expected lifetime remaining estimated for the respondent. All subsequent TTOs were based on the response to this question, as outlined in Figure S1.

Figure S3. Average difference in scores (1=never, 5=always) between the time of the reported individual highest and lowest HbA_1c_ for three groups of people with diabetes having experienced different changes in HbA_1c_

Significant association between change in the frequency of experiencing excessive thirst and change in HbA1c (p<0.0001). Error bars denote 95% confidence intervals.

Figure S4. Average difference in scores (1=never, 5=always) between the time of the reported individual highest and lowest HbA1c for three groups of people with diabetes having experienced different changes in HbA_1c_

Error bars denote 95% confidence intervals.

Figure S5. Average difference in scores (1=never, 5=always) between the time of the reported individual highest and lowest HbA_1c_ for three groups of people with diabetes having experienced different changes in HbA_1c_

Significant association between change in the frequency of experiencing frequent urination and change in HbA_1c_ (p<0.0001, p=0.0005 for day and night respectively). Error bars denote 95% confidence intervals.

Figure S6. Average difference in scores (1=never, 5=always) between the time of the reported individual highest and lowest HbA1c for three groups of people with diabetes having experienced different changes in HbA_1c_

Significant association between change in the frequency of experiencing fatigue symptoms and change in HbA_1c_ (p<0.0001 and p=0.0001 for tiredness/lack of energy and fatigue/extreme tiredness respectively). Error bars denote 95% confidence intervals.

Figure S7. Average difference in scores (1=never, 5=always) between the time of the reported individual highest and lowest HbA1c for three groups of people with diabetes having experienced different changes in HbA1c

Significant association between feeling “scared…” about living with diabetes and a change in HbA_1c_ (p=0.0328). Error bars denote 95% confidence intervals.

Figure S8. Average difference in scores (1=never, 5=always) between the time of the reported individual highest and lowest HbA1c for three groups of people with diabetes having experienced different changes in HbA1c

The p value for the association between “feeling depressed…” and change in HbA_1c_ was p=0.06 (not significant). Error bars denote 95% confidence intervals.
